# Ten years of oil pollution detection in the Eastern Mediterranean shipping lanes opposite the Egyptian coast using remote sensing techniques

**DOI:** 10.1038/s41598-024-67983-x

**Published:** 2024-08-05

**Authors:** Salma M. Baghdady, Ali A. Abdelsalam

**Affiliations:** 1 Biological, Marine and Agriculture Environmental Sciences Department, Environmental Studies Institute, Arish University, El-Arish, Egypt; 2 Department of Marine Science, National Authority for Remote Sensing and Space Sciences (NARSS), Cairo, Egypt

**Keywords:** Mediterranean Sea, SAR, Marine pollution, Maritime traffic, Remote sensing, Illegal discharges, Environmental sciences, Ocean sciences, Physics

## Abstract

The Eastern Mediterranean region, a vital conduit for global maritime trade, faces significant environmental challenges due to marine pollution, particularly from oil spills. This is the first study covering the long period of comprehensive monitoring of oil pollution using the full mission of Sentinel-1 Synthetic Aperture Radar (SAR) data in the Mediterranean Sea, so this research aims to detect and analyze comprehensively the occurrence of oil spills in the Eastern Mediterranean over a decade (2014–2023). This study focuses on identifying geographical distribution patterns, proximity to shorelines, frequency across maritime zones, and potential sources of these spills, especially around major ports and maritime routes. This study utilizes SAR data from the Sentinel-1 satellite. The methodology included automated detection algorithms within the Sentinel application platform (SNAP) and integration with GIS mapping to study oil spill patterns and characteristics. Over 1000 Sentinel-1 scenes were investigated in the northern Mediterranean waters off the coast of Egypt, to detect and analyze 355 oil spill events with a total impacted area of more than 6000 km^2^. The analysis of temporal spill distribution reveals significant fluctuations from year to year. Within the entire timeline of the study, 2017 had the largest spatial areas covering one thousand square kilometers. In contrast, the single largest spill recorded during the study period occurred in 2020, covering 198.73 square kilometers. The results identified a non-uniform distribution of oil spills and primarily exhibiting elongated patterns aligned with the navigation routes. The distinct increase of oil spill incidents was within the Exclusive Economic Zone (EEZ), obviously drifted to the coastline and around major ports. The study emphasizes the critical role of remote sensing technologies in addressing environmental challenges caused by the maritime transport sector, advocating for enhanced monitoring and regulatory enforcement to protect marine ecosystems and support sustainable naval activities. The findings highlight the urgent need for targeted continuous monitoring and rapid response strategies in high-traffic maritime areas, particularly around the EEZ and major ports.

## Introduction

The Eastern Mediterranean possesses immense strategic importance due to its crucial role in facilitating global trade. This is notable through the heavily trafficked Suez Canal, which is responsible for approximately 12% of global trade and 30% of worldwide container traffic, according to the Suez Canal Authority (2022). However, this increased maritime activity poses significant challenges, including the risk of marine pollution, especially from oil spills, which adversely affect fragile marine ecosystems. Consequently, the region's marine traffic represents global connectivity and environmental vulnerability, necessitating prudent management to protect the marine ecosystem.

Oil spills in the Mediterranean Sea, a heavily trafficked maritime region, are primarily caused by tanker accidents, operational discharges, and illegal dumping from ships. These incidents release vast quantities of petroleum hydrocarbons into the marine environment, leading to severe ecological and economic repercussions. The Mediterranean's enclosed nature exacerbates the persistence of oil, which significantly impacts marine life and habitats^1^. Immediate effects include smothering and toxicity to marine organisms such as fish, seabirds, and marine mammals, leading to widespread mortality. Chronic impacts are equally alarming, as oil residues settle into sediments, disrupting benthic habitats and biogeochemical cycles over extended periods^2^. Economically, the fishing and tourism industries suffer substantial losses. Contaminated fish stocks force fishery closures, altering local economies and livelihoods. Tourism, a major economic driver in the region, faces declines as oil-polluted beaches deter visitors and harm the image of pristine Mediterranean destinations^3^. Operational discharges from ships, specifically bilgewater, raise environmental concerns. This black liquid mixture, containing oil, water, sludge, and chemicals, can have detrimental effects on marine environments^4^. To mitigate pollution, bilge waters should be processed on board using oily water separators (OWS) to ensure oil content meets the compliance levels set by international regulations^5^. Bilge waters must also be periodically removed and transferred to the nearest port facility^6^. However, for economic reasons, some ship operators may illegally discharge oily waste at sea, leading to severe environmental damage^7–9^. These discharges can disrupt ecosystems, damage fisheries and tourism, and pose health risks^10,11^. The Mediterranean Sea, designated as a special area under MARPOL 73/78 ANNEX I, prohibits the discharge of oily mixtures or oil effluents into the sea, except when the oil content is below 15 PPM^5^ a concentration undetectable by SAR imagery^12^. Despite these regulations, some vessels continue to discharge bilgewater illegally, particularly at night, which exacerbates the problem of marine pollution in the Mediterranean.

Remote sensing technology, especially SAR imagery, is crucial for detecting oil pollution, operating day and night, and covering vast areas^13,14^. In high-traffic regions like the Mediterranean, spaceborne surveillance is essential for rapid detection, clean-up, and identifying polluters^15,16^.

Monitoring oil spills in the Mediterranean Sea using remote sensing has evolved significantly over the decades, with advancements in technology and data availability enhancing detection capabilities. Early efforts in the 1980s and 1990s primarily relied on optical sensors and sporadic satellite imagery, providing limited temporal coverage. The launch of the European Space Agency's ENVISAT satellite in 2002 marked a significant milestone, offering regular SAR data, which proved crucial for consistently monitoring oil spills^17^. The introduction of the Sentinel-1 satellites in 2014 further revolutionized oil spill detection, providing high-resolution, dual-polarization SAR imagery with frequent revisit times, allowing for near real-time monitoring^16,18^. Over the years, studies have utilized these datasets to analyze oil spill patterns, distribution, and sources comprehensively. They highlighted the critical role of remote sensing in environmental monitoring and maritime safety in the Mediterranean region^19–21^.

Many studies indicate a significant portion of annual oil pollution in oceans comes from fuels and crude oil, with intentional oil spills from operational discharges occurring more frequently than accidents^14,22,23^. Also, some Studies have demonstrated the impact of oil slicks on sea surfaces, with radar imaging measurements, providing an in-depth analysis of oil spill detection through satellite remote sensing on sea surfaces with wind speeds below 2 m/s and above 15 m/s^13^. Furthermore, according to Abou Samra and Ali^24^, the analysis of Sentinel-1 data of the incident showed oil slick accumulation along the Syrian coast for several days. Moreover, the study identified wind speed and direction as crucial factors in the dispersion of the spilled oil. During their study, Ferraro et al.^17^ conducted a comprehensive mapping of oil spills in the Mediterranean Sea, identifying 150 potential oil spill events and demonstrating the effectiveness of remote sensing techniques for environmental monitoring and the necessity for continuous surveillance to protect marine ecosystems. Similarly, Abou Samra et al.^25^ used the Environmental Sensitivity Index (ESI) to assess oil spill impacts along the Nile Delta coast, identifying high-risk oil spill areas. Moreover, recent studies using SAR data and remote sensing techniques have accurately detected and mapped oil spills in the Mediterranean, highlighting the need for integrated data and coordinated response strategies^23,26–29^.

This study aims to comprehensively analyze oil spills in the Eastern Mediterranean over a ten-year period from 2014 to 2023, providing an extensive and up-to-date dataset. The novelty of this research lies in its in-depth examination of the geographical distribution of spills, their proximity to the shoreline, and their frequency across different maritime zones, offering a nuanced understanding of the issue. Additionally, it identifies potential sources of oil spills around major ports and shipping routes, contributing valuable insights into specific areas of concern.

## Methodology

### Study area

**Figure 1:**
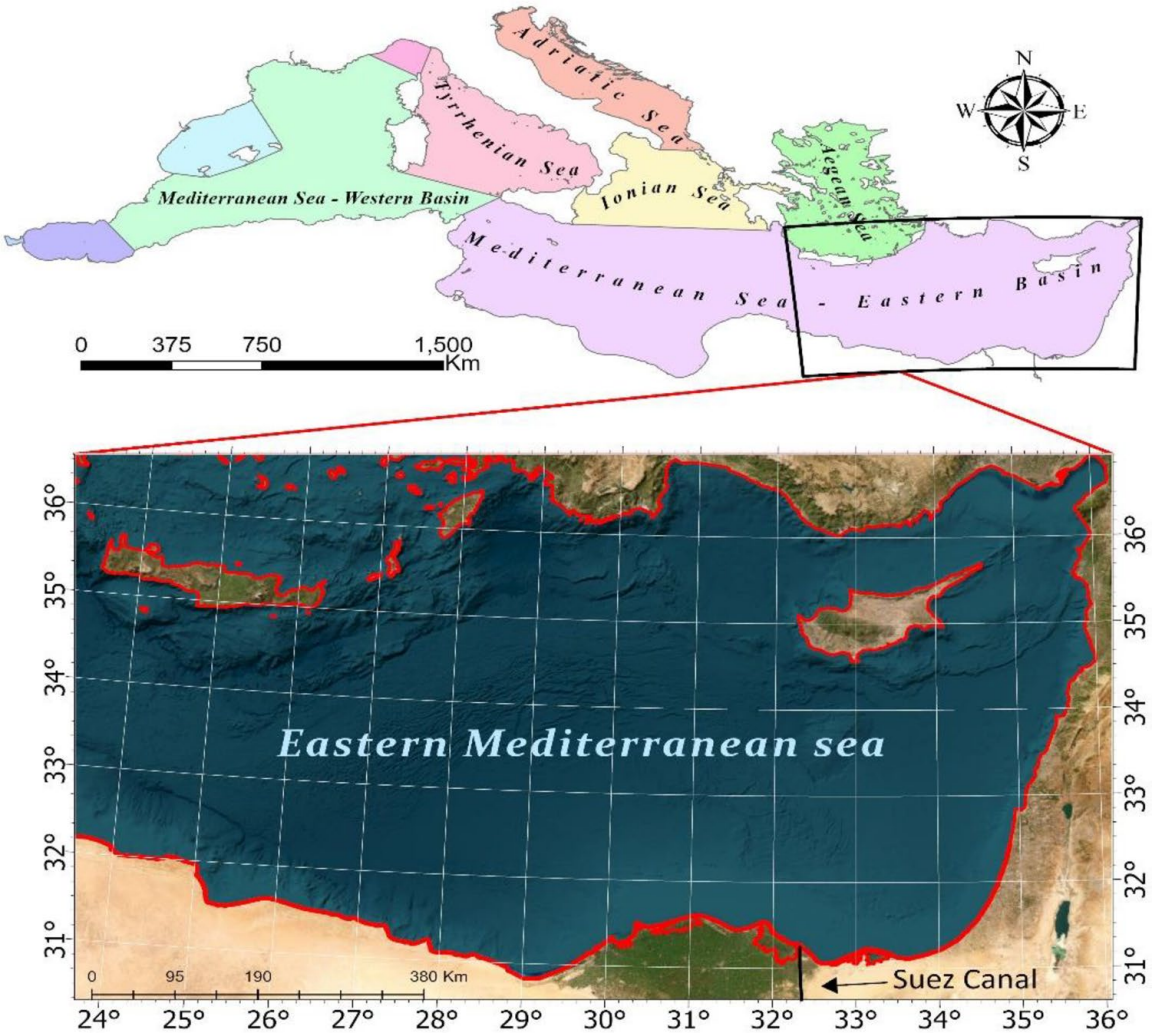
The study area of the Levantine basin.

**Figure 2:**
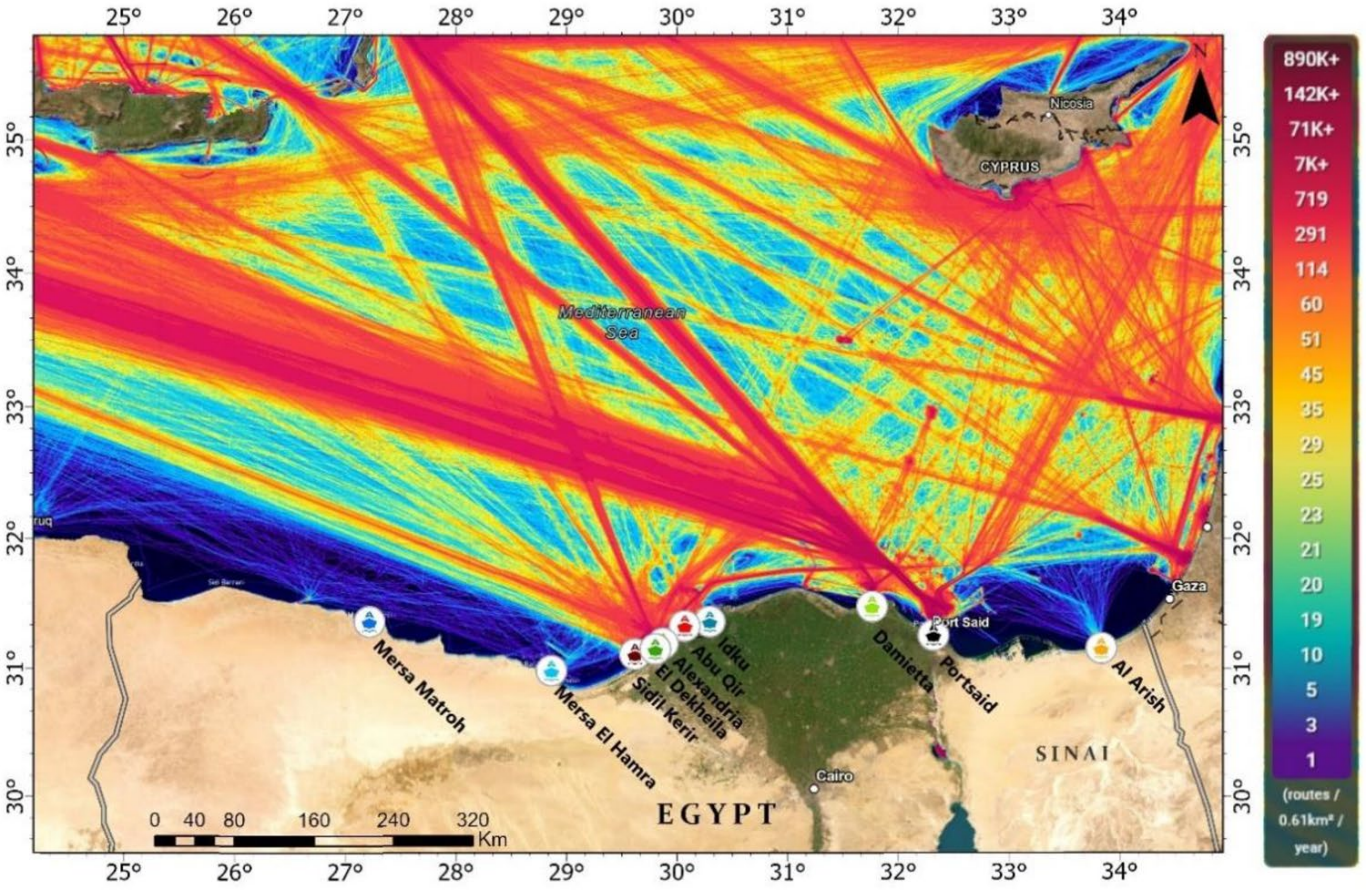
Showing marine traffic and Egyptian ports located in the study area.

The study area is located along the North coast of Egypt in the Eastern Mediterranean (Fig. 1) and encompasses several strategically important ports (Fig. 2), including Port Said, Alexandria, Idku liquefied natural gas (LNG) Port, Abu Qir Port, Damietta Port, El-Dekheila Port, and Sidi Kirir Terminal. Due to their strategic geographical positioning, these ports play a critical role in global maritime trade, serving as key junctions connecting Europe, Africa, and Asia. Port Said, positioned at the northern entrance of the Suez Canal, is particularly significant, as it channels approximately 12% of global trade through this vital access point, linking the North Atlantic Ocean to the northern Indian Ocean. Egypt's principal port, Alexandria, handles the majority of the nation's foreign trade, Damietta Port, known for its extensive area and various terminals, located west of the Nile's Damietta branch. while Idku LNG Port is the largest LNG export facility in the country. These ports have indispensable role in the international maritime commerce network and contribute significantly to Egypt's economy by enhancing trade efficiency, generating employment, and stimulating related sectors such as shipping and logistics. Given the high volume of vessel traffic through this area, it is plausible to suggest that this region could be susceptible to oil spills. The prevalence of such incidents could pose severe environmental threats to the marine ecosystem. Therefore, the selection of this region for the study is driven by its strategic importance, heavy maritime traffic, and the associated risks of marine pollution, especially from oil spills. The prevalence of oil spill incidents in this area underscores the need for comprehensive monitoring and effective response strategies to protect the marine environment.

The Eastern Mediterranean, specifically along the Egyptian coast, features a Mediterranean climate with summer temperatures ranging from 25 to 35 °C and winter temperatures between 10 and 20 °C. Wind patterns are dominated by the northerly Etesian winds, with speeds generally between 2 to 10 m/s, influencing oil spill dispersion by pushing spills southward along the coast. The area receives minimal rainfall, averaging 100 mm to 400 mm annually, mostly concentrated from November to March, which minimally affects oil spill behavior. Wave heights typically remain below one meter but can exceed two meters during storms, significantly mixing oil into the water column. Relative humidity fluctuates from over 65% in winter to around 55–60% in summer, with high solar radiation enhancing the evaporation of oil during warmer months. These conditions crucially impact the spread, behavior, and management of oil spills in this region.

### Remote sensing data

The SAR data used in this study was sourced from the Sentinel-1 satellite, which is equipped with a C-band synthetic-aperture radar instrument. This type of radar sensor is considered an active sensor. It operates in the C-band wavelength, which enables it to penetrate clouds and acquire data during the day and night, making it an essential tool for earth observation and marine monitoring.

In this study, Sentinel-1 data used were utilized in Ground Range Detected (GRD) level 1 data format, with a dual-polarization mode. The data acquisition mode was Interferometric Wide Swath (IW), which provides a 5 × 20-m spatial resolution and a 250 km swath width. The incidence angle range for the SAR data was between 29.1° and 46.0°. To perform the study, numerous SAR scenes from the study area were examined over a period of ten years, from 2014 to 2023. The data were obtained from the European Space Agency—ESA Sentinel data hub, which provides free access to Sentinel-1 data. Wind speed data were also acquired from NASA's Modern-Era Retrospective Analysis for Research and Applications, Version 2 (MERRA-2).

### Automated detection of oil spill

**Figure 3:**
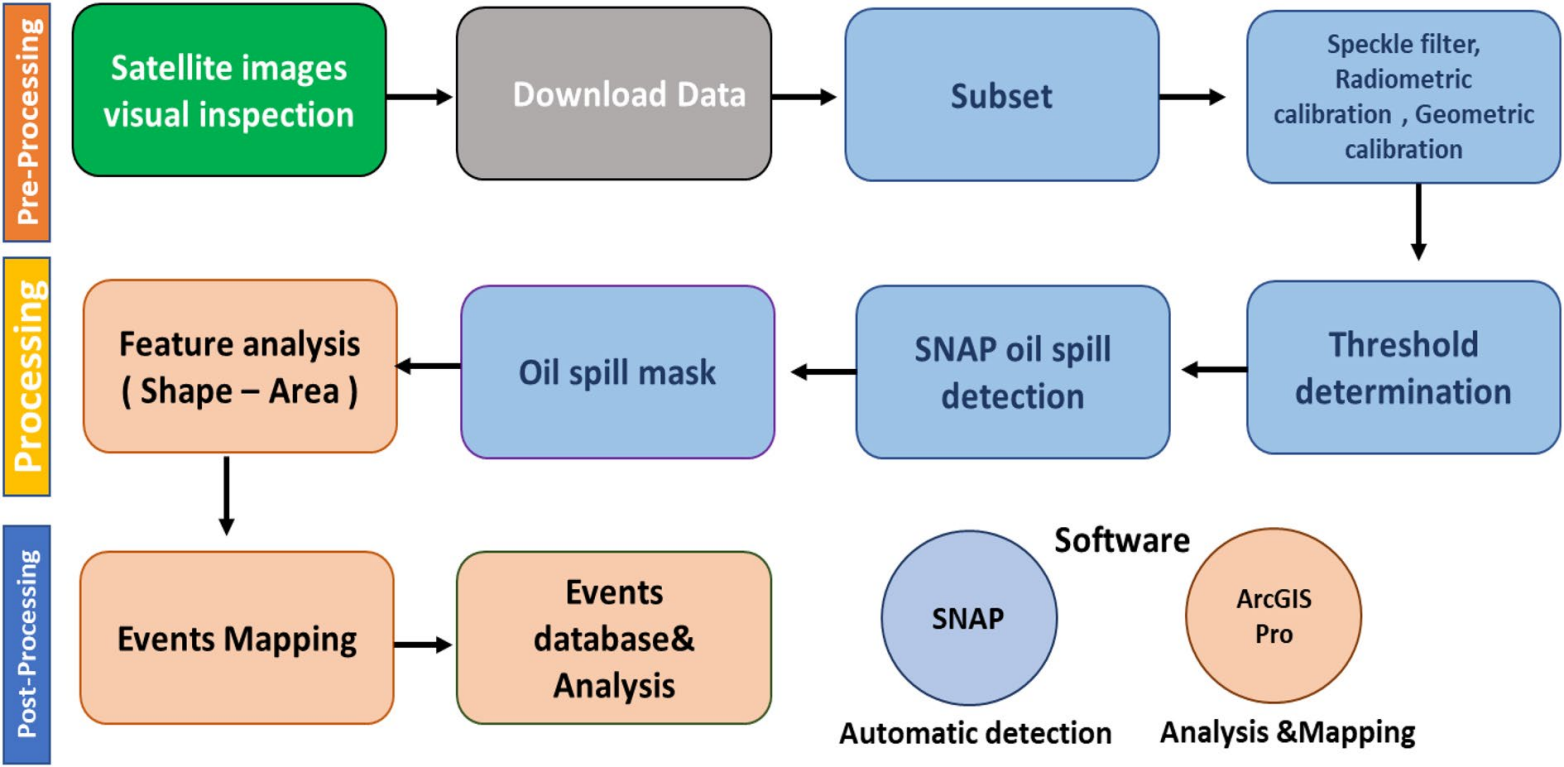
Flowchart methodology of automated detection of oil spill.

**Figure 4:**
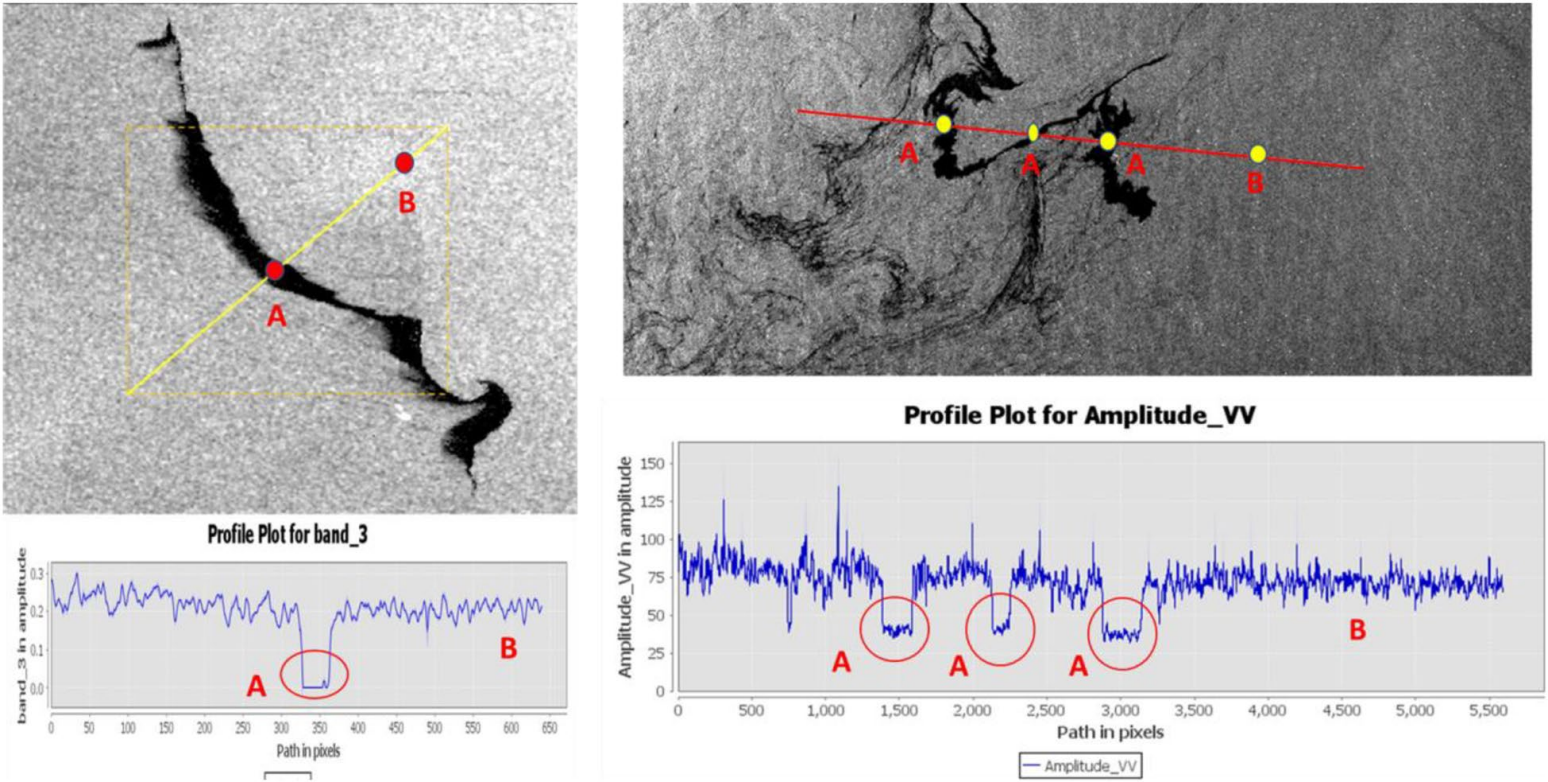
Profile plot of SAR-C Sentinel-1A image of oil spill detected, (**A**) the dark spot Region presents the oil spill. (**B**) Presents Normal Sea water reflectance.

**Figure 5:**
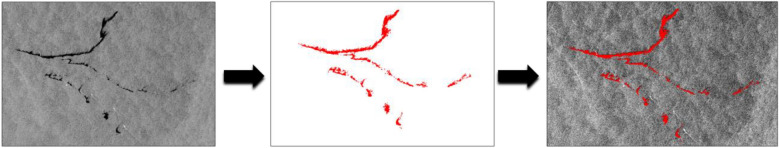
SAR-C Sentinel-1A image of oil spill northwestward Suez Canal entrance on 26/12/2021. Left: the preprocessed raw data, Middle: the raster oil spill mask to calculate area and geographical location and Right: visualized the oil spill mask on the preprocessed raw data.

To detect the oil spills, the SNAP Graph Builder (Fig. 3) was utilized to create a processing graph tailored to the specific requirements of this study. The graph incorporated various processing steps, including radiometric calibration, multi looking, speckle filtering, threshold detection (Fig. 4) and oil spill detection algorithms. Multi-looking was applied to reduce speckle noise by averaging multiple looks, thereby enhancing image quality. Speckle filtering, specifically the Lee filter with a 7 × 7 window size, was used to further reduce noise while preserving edges and fine details critical for accurate oil spill detection. The core of our methodology was the oil spill detection algorithm, which was integrated into the processing graph. This algorithm utilized the radar backscatter characteristics of oil spills to distinguish them from the surrounding sea surface. An adaptive thresholding technique was applied to identify oil spill events. We analyzed the profile plots depicted in the provided SAR imageries (Fig. 3). Lower amplitude values typically indicate areas of oil spills, appearing as darker regions in SAR imagery due to their reduced backscatter. In the profile plots, regions marked as 'A' correspond to dips in amplitude, indicative of low backscatter values, while regions marked as 'B' signify higher backscatter. These identified low amplitude ranges align with the regions marked as 'A' on the images, indicating potential oil spills where the backscatter values are significantly reduced due to the presence of oil on the water surface. Upon completion of the processing steps, the results were analyzed in ArcGIS Pro to study and map the areas affected by oil spills, distance from the shoreline, and the frequency and intensity of oil spills (Fig. 5). This methodology provided valuable insights into the spatial and temporal distribution of oil spills in the Eastern Mediterranean over the ten-year period.

### Weathering conditions

Wind speed data can substantially improve the discrimination of oil spills from look-alikes. By analyzing wind speeds, we can better understand surface conditions, which helps accurately detect oil spills on the sea surface. The most appropriate SAR configuration for slick detection is C-band (3.75–7.5 cm) single-polarized VV SAR at incidence angles of 20° to 45°. Wind speeds must be between 2 and 14 ms^−1^ for an oil spill to be detectable.

## Results

### Spatial analysis

**Figure 6:**
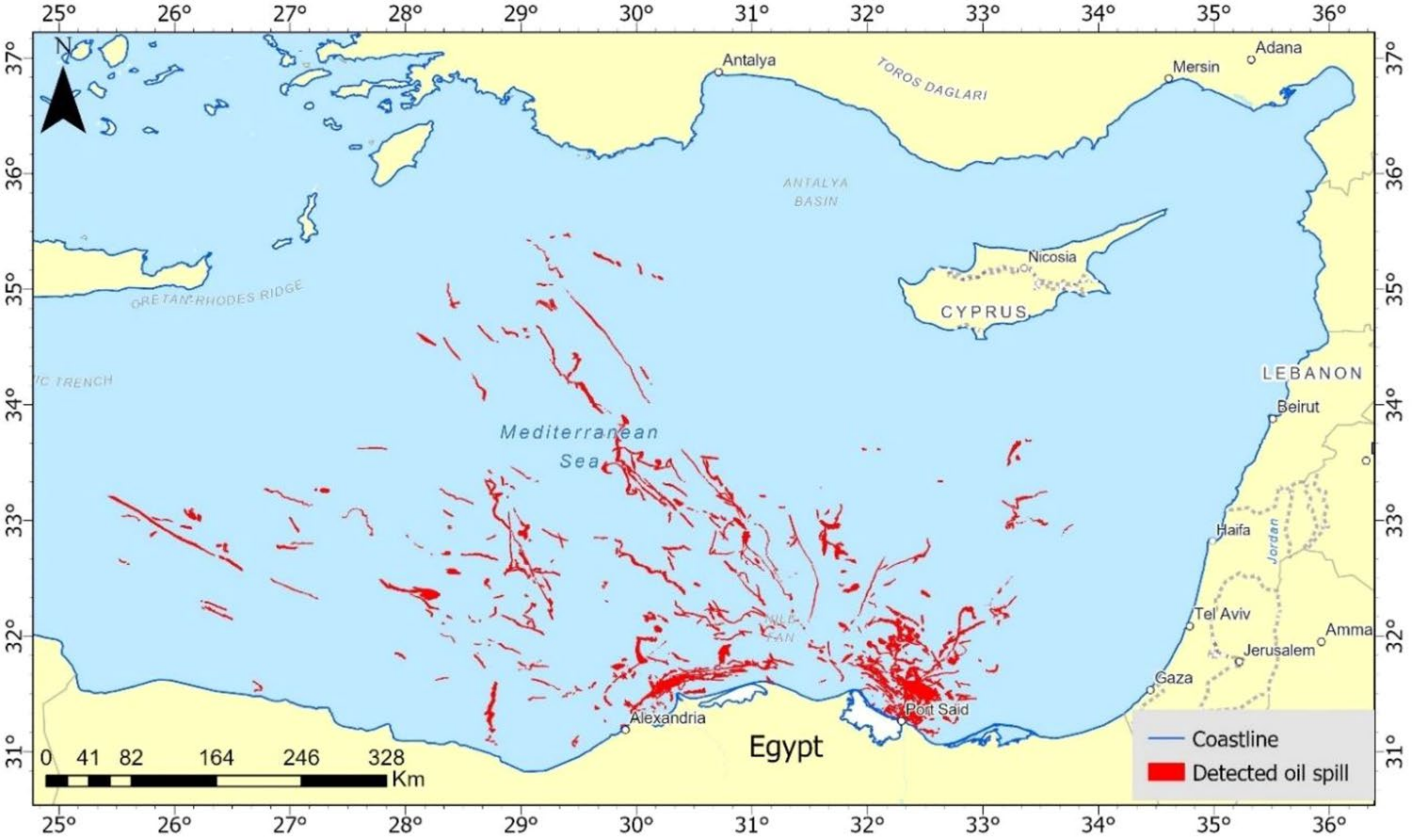
Accumulative map of oil spills in the Eastern Mediterranean over ten years from 2014 to 2023.

Over the ten years, from 2014 to 2023, around a thousand Sentinel-1 images were examined to record 355 events detected from highly suspected scenes. The geographical distribution of oil spills (Fig. 6) explains and shows scattered patterns across the Eastern Mediterranean Sea, with a clear concentration along the Egyptian coastline. This distribution is non-uniform, with some areas experiencing a denser accumulation of spills. The pattern extends from the Nile Delta region near Alexandria and flows out to the northeastern direction, towards the Levantine Sea.

Some of the densest clusters of oil spills are found off the coast of major Egyptian cities such as Alexandria, Damietta and Port Said. Moving eastward, the incidence of spills appears to thin out but is still present along the maritime paths leading to and from the Suez Canal. As depicted on the provided map, the distribution patterns of oil spills in the Eastern Mediterranean Sea, indicate a concentration of incidents clustered around major maritime routes and proximal to the Egyptian coastline.

#### Distance from shoreline

**Figure 7:**
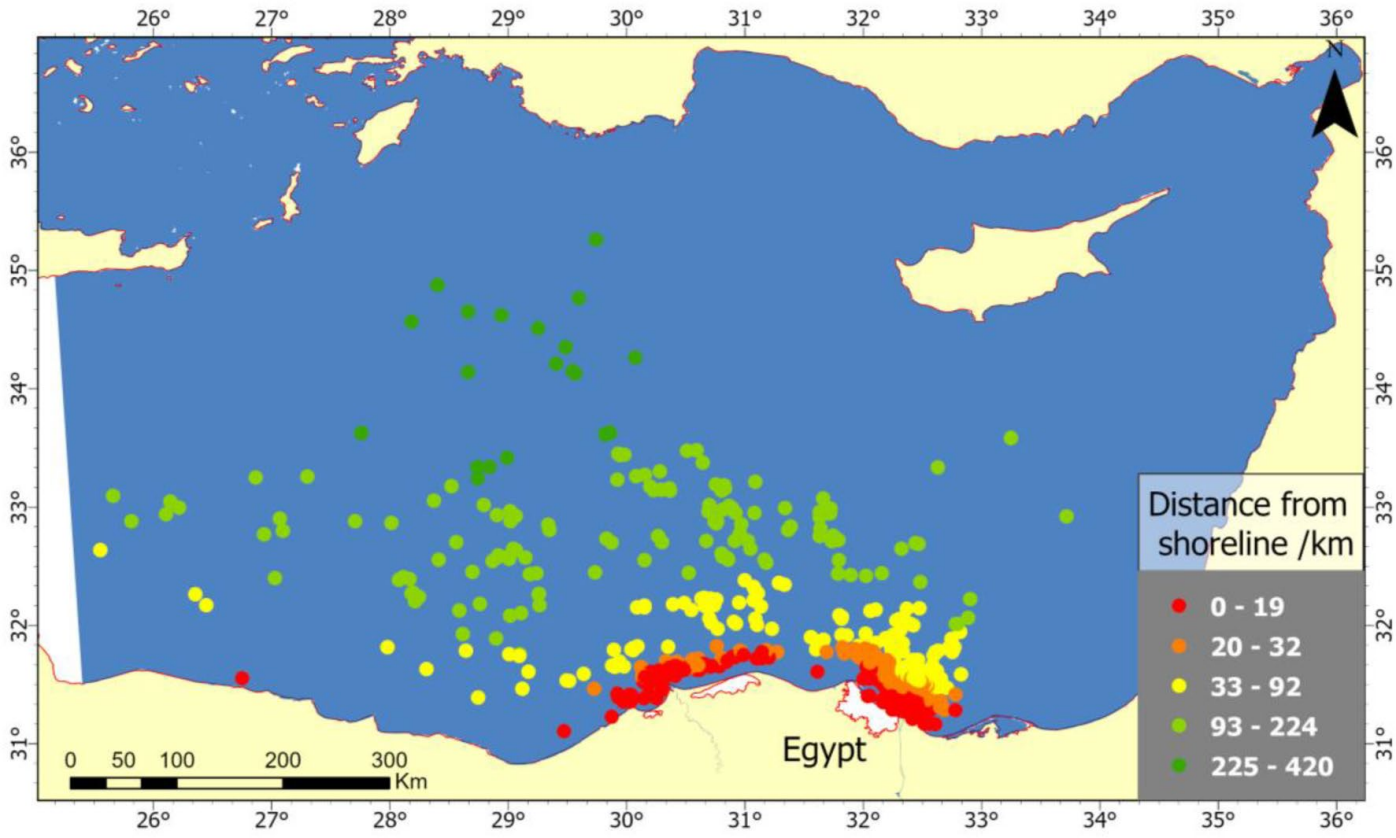
Geographical distribution map of oil spills over ten years according to its distance from the shoreline.

The distribution of oil spills, as depicted on the map (Fig. 7), which clearly shows their proximity to the shoreline. The most frequent occurrences are situated within 19 km of the coastline. The dense clustering along the coast highlights zones of heightened spill activity.

As the distance from the shoreline increases, the frequency of spills appears to decline. The spread of the second class, representing the 20–92 km range from the shoreline, shows a noticeable dispersal pattern into the sea. Moving further away from the coast, in the 93–420 km range, the 4 and 5 classes illustrate a more sparse and scattered distribution of oil spills.

#### Analysis according to maritime zones

**Figure 8:**
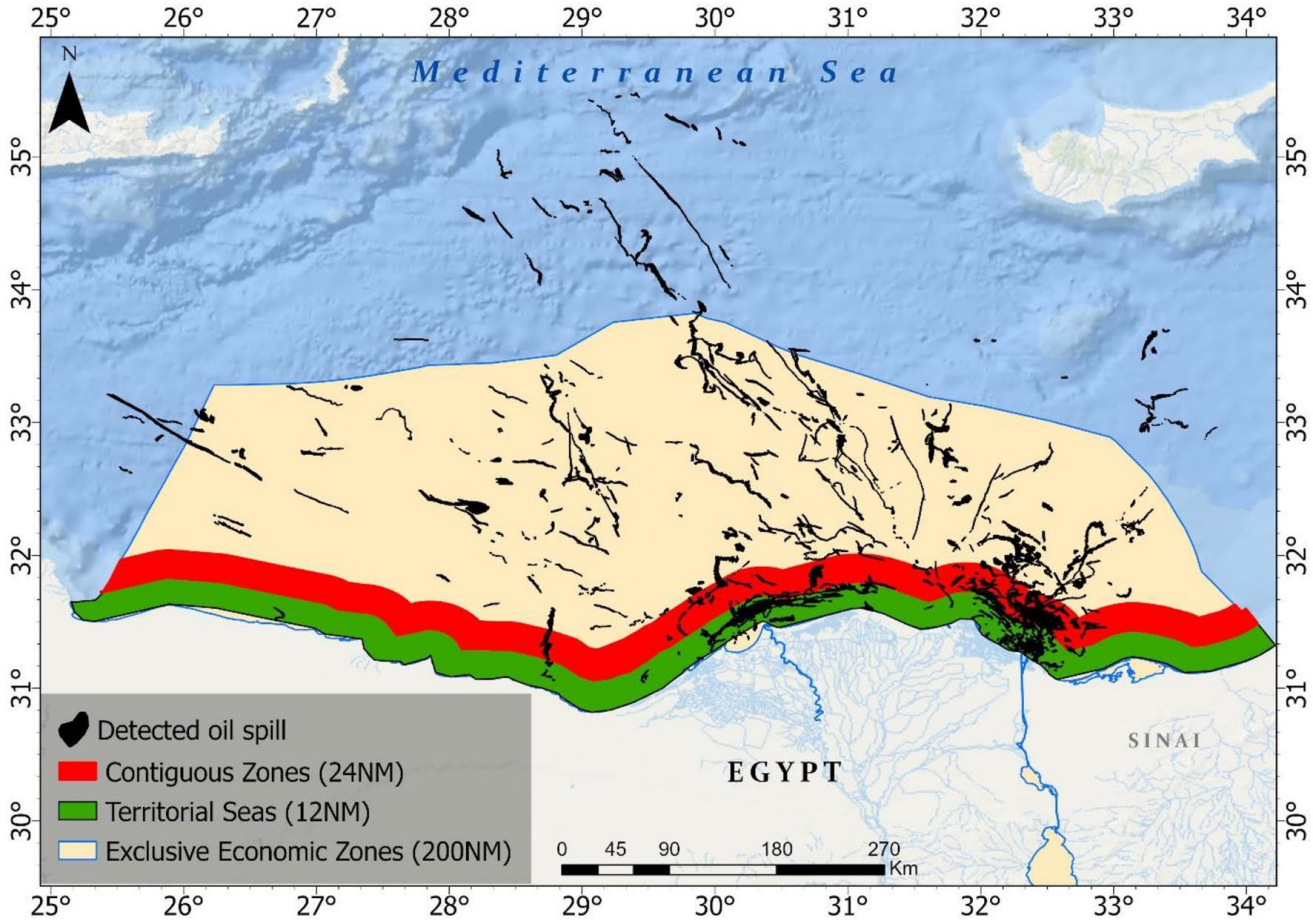
Showing the spatial distribution of the detected oil over 10 years about Egyptian Maritime Zones.

The spatial analysis of the detected oil spills over a ten-year period shows a distinct variation in the frequency of events across different maritime zones adjacent to the Egyptian coastline (Fig. 8). Within the Territorial Zone, which extends up to 12 nautical miles from the coastline, there were 91 recorded oil spill events. These spills were generally smaller in the area compared to those in the more distal maritime zones.

Moving outward from the coast, the Contiguous Zone, extended from 12 to 24 nautical miles from the coastline, exhibited a slightly higher incidence, with 103 oil spill events detected. Furthermore, from the coast, the EEZ, reaching up to 200 nautical miles offshore, the data indicates a substantial increase in spill incidents with 161 detected spills. The oil spills detected in this area were more dispersed across the eastern Mediterranean with mostly longitudinal patterns compared to the oil spills detected in the other zones.

### Temporal analysis

#### Annual analysis

**Figure 9:**
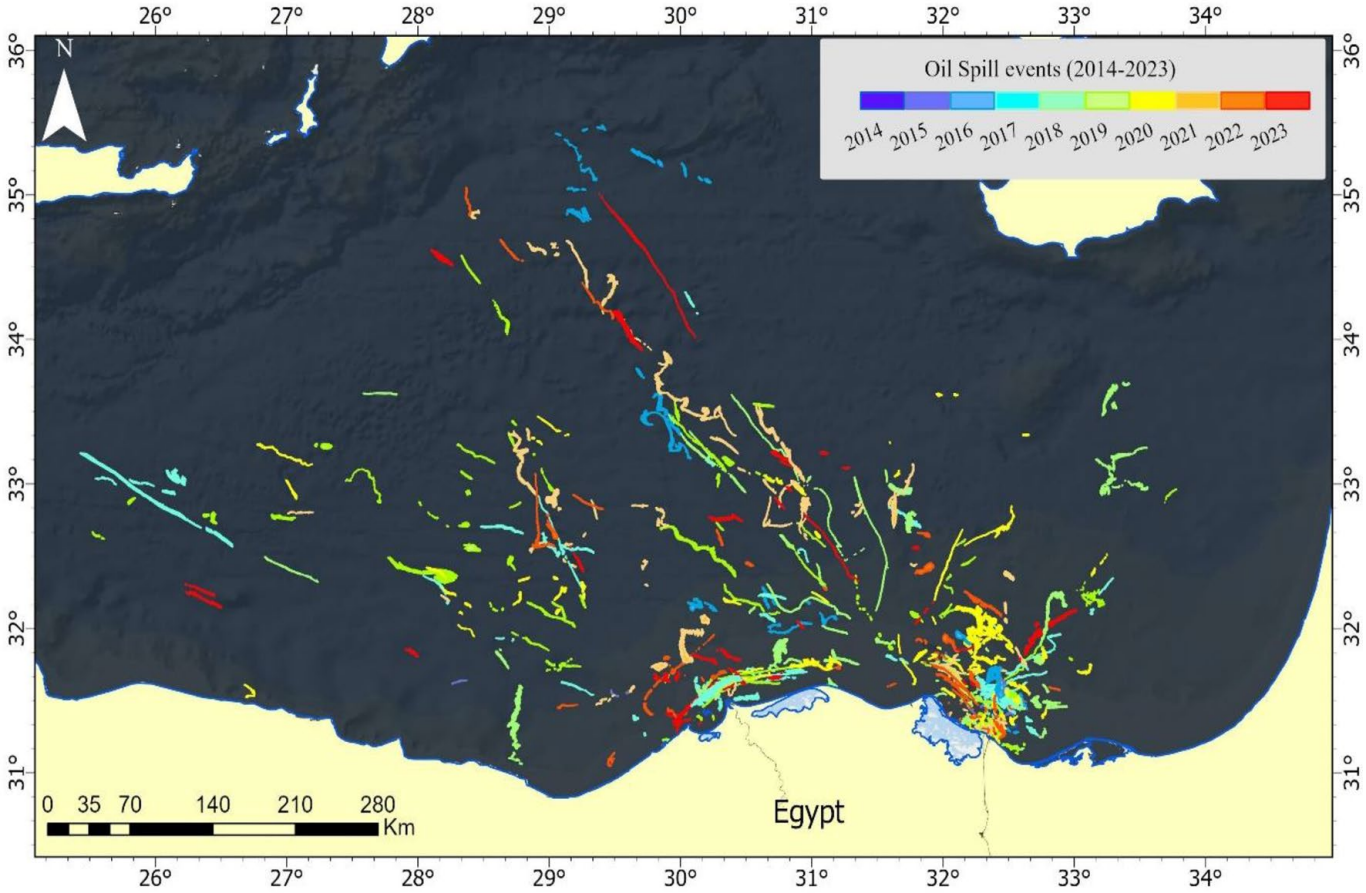
Map presenting the distribution of oil spills detected from 2014 to 2023 each year.

**Figure 10:**
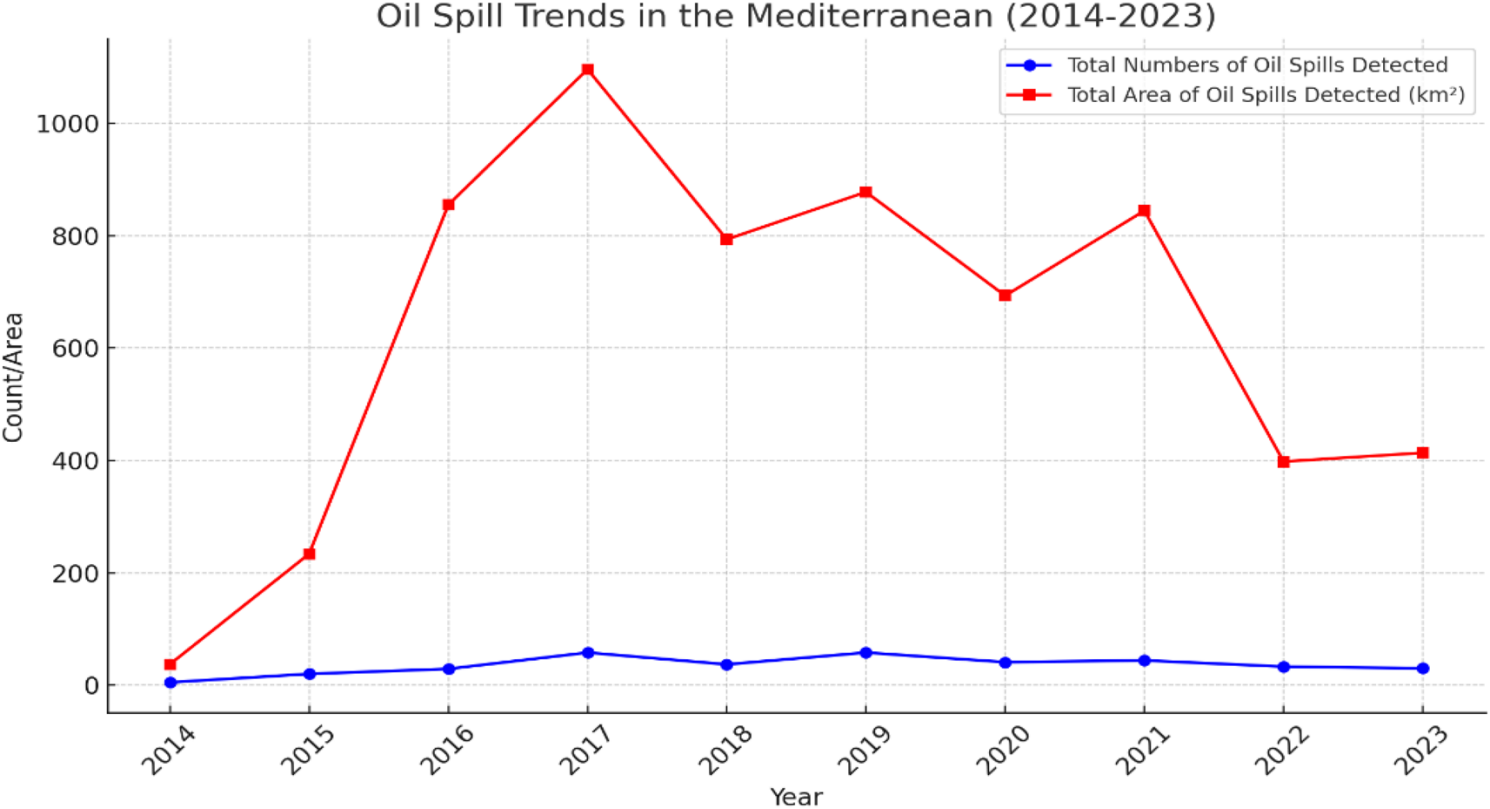
The graph presents the total number and area of oil spills detected yearly over a decade.

The comprehensive analysis of oil spill data in the Mediterranean Sea from 2014 to 2023 underscores significant variability in both the incidence and the total area (Fig. 9). Over this decade, the cumulative record indicates 355 oil spills, affecting a substantial total area of 6244.14 km^2^. The years 2017 and 2019 emerged as particularly noteworthy, each recording 58 spills, and 2017 marked the peak in spatial impact with a 1096.42 km^2^ area affected. This (Fig. 10) notably exceeded the spill areas in 2016 (855.26 km^2^) and 2019 (877.92 km^2^). Furthermore, 2016 was characterized by a higher mean area per spill, averaging 29.49 km^2^, suggesting that spills in this year, though fewer, were larger on average. In contrast, 2014 and 2022 witnessed the lowest total affected areas, with 37.27 km^2^ and 398.10 km^2^ respectively.

#### Seasonal and monthly analysis

**Figure 11:**
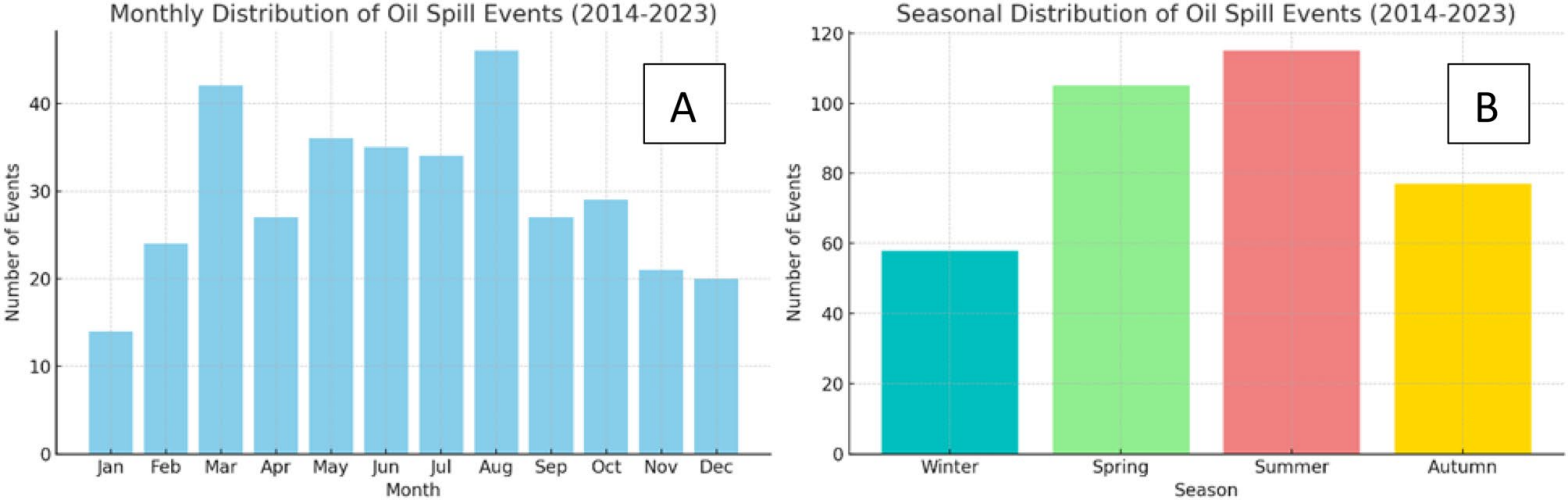
Shows the monthly and seasonal distribution of oil spill events detected over ten years (**A**) represents monthly data, and (**B**) represents the seasonal data.

The monthly and seasonal analysis of oil spill events reveals a clear seasonal trend in oil spill occurrences (Fig. 11B). The summer season is observed to have the highest incidence, with 115 recorded events, while spring follows with 105 events. These two seasons combined account for a majority of the annual oil spills. Conversely, autumn shows a reduction in numbers to 77 events, and winter has the fewest at 58 events.

On a monthly scale (Fig. 11A), The monthly distribution shows variability throughout the years. The highest number of events was detected in August, while January saw the lowest. The data demonstrate a gradual increase in oil spill events commencing in March, peaking in August, and declining towards the year's end.

### Oil spill events analysis

#### Spill area variations

**Figure 12:**
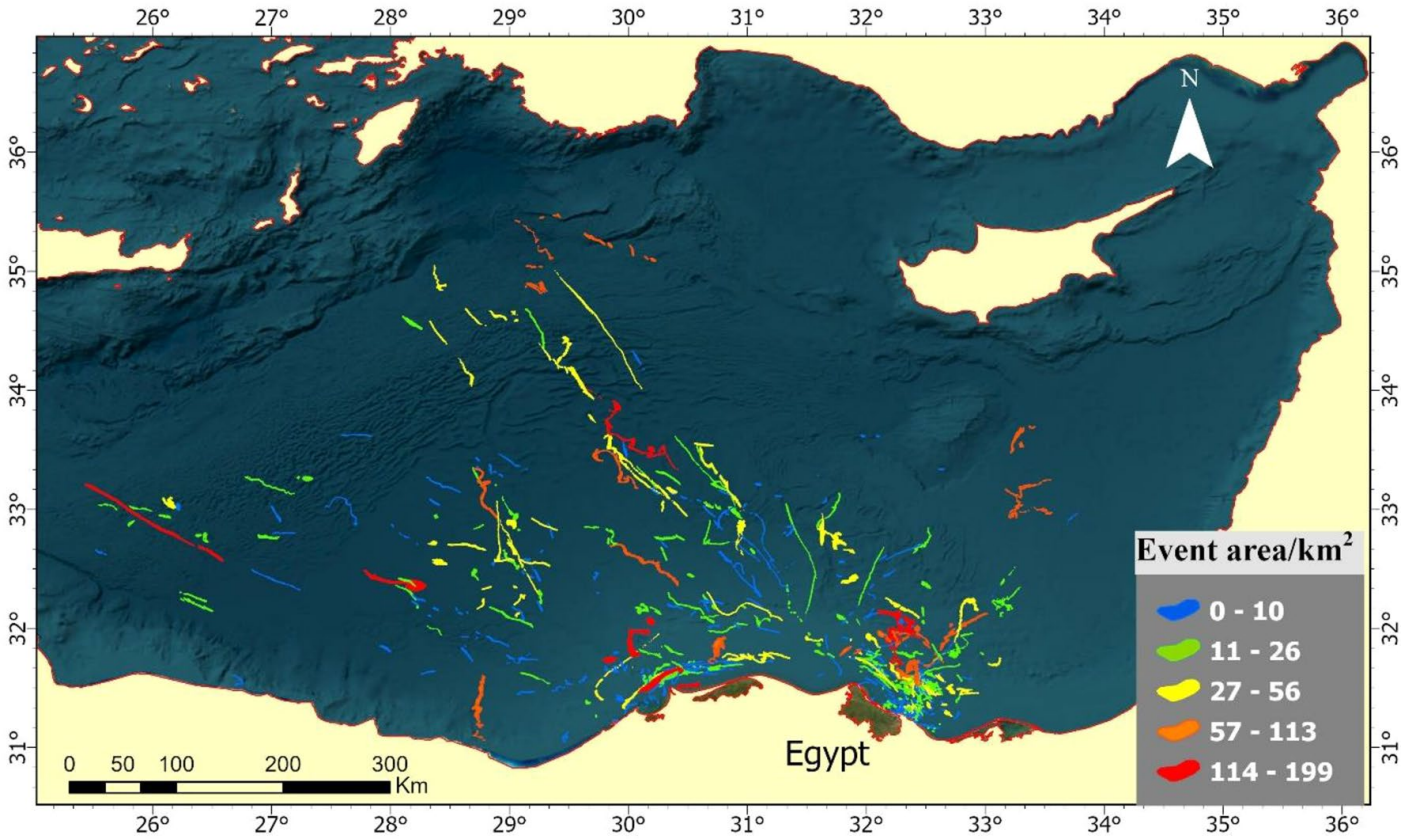
Map of oil spill event areas in the Eastern Mediterranean: a 10-year area analysis.

The areas affected by oil spills, depicted in Fig. 12, show a notable rise in the frequency of smaller-scale spills, with 191 events covering areas less than 10 square kilometers. These spills are widely dispersed across the marine area and do not show a significant concentration in any particular region. This is followed by spills of intermediate sizes, which exhibit a varied distribution, with some clustering along the coast and others dispersed further offshore. In contrast, the largest spills, of which there were only five, show a significant decrease in frequency but are distributed throughout the entire region.

**Figure 13:**
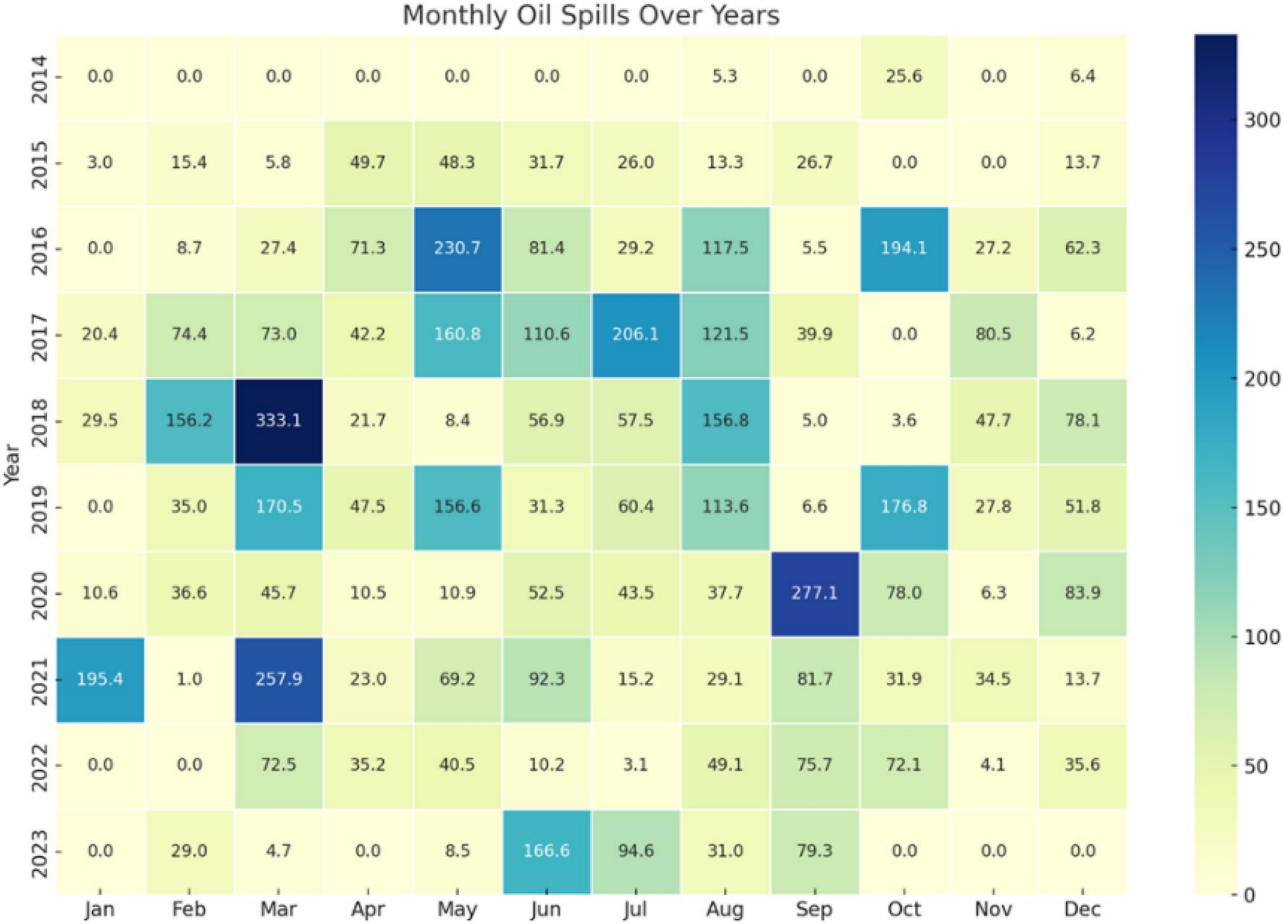
The heatmap provides a color-coded oil spills for each month across 10 years.

The monthly events areas analysis, covering a decade-long detection period, shows that March had the largest cumulative area affected by oil spills, approximately 990.61 km^2^, followed by May and August with 734 km^2^ and 674.82 km^2^, respectively (Fig. 13). In contrast, November recorded the smallest affected area, with 228.13 km^2^ of detected oil spills.

#### Oil spill events frequency

**Figure 14:**
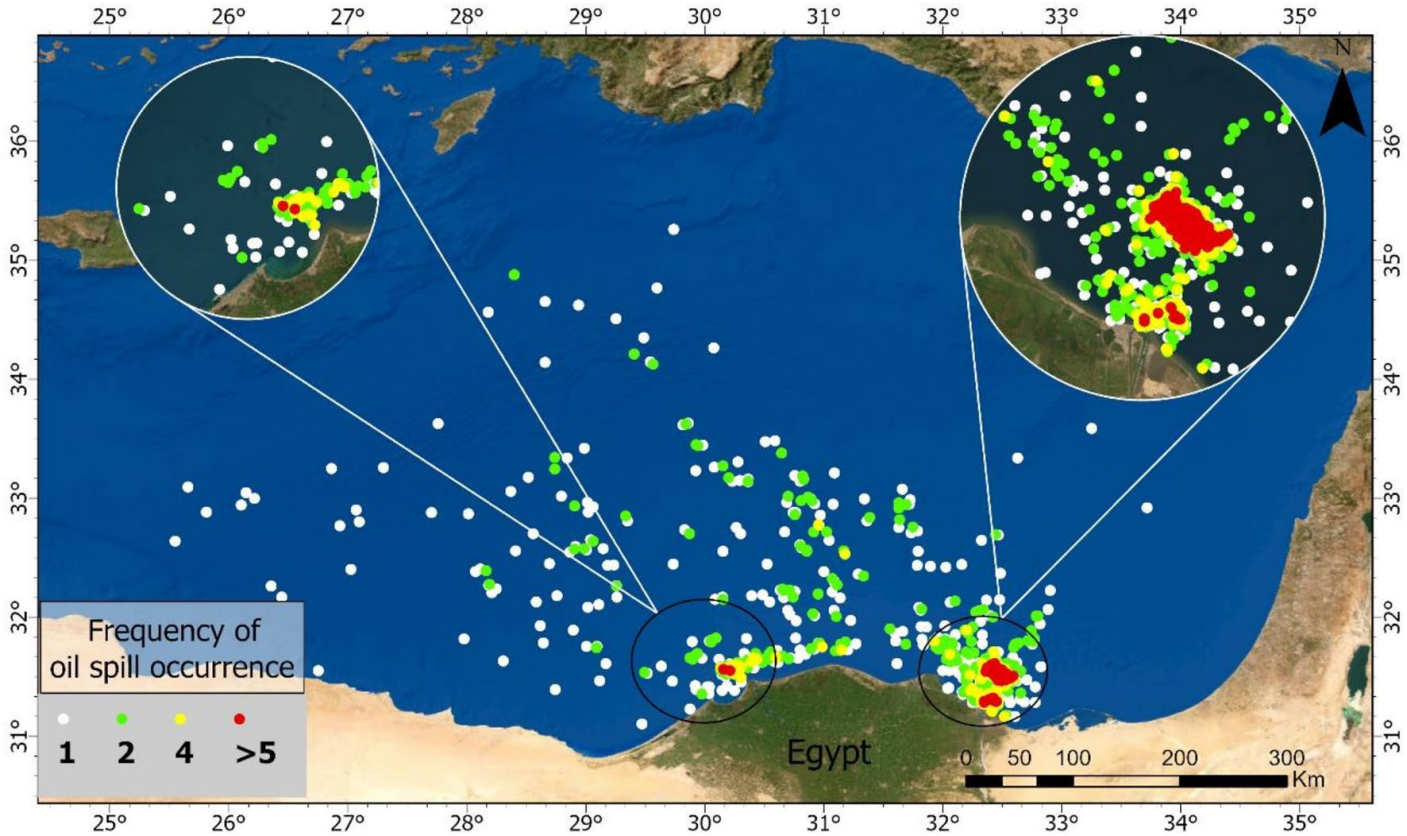
The frequency of oil spills detected in the eastern Mediterranean over 10 years.

A high frequency of oil spills, indicated by 5- time occurrence, appears to be concentrated notably in close proximity to the Nile Delta and along the Suez Canal (Fig. 14). These areas are marked by zoomed-in circles, highlighting the regions with the densest occurrence of detected oil spills from 2 to 3-time occurrences, representing lower frequencies of oil spills, are spread more evenly across the study area, with a noticeable distribution along the eastern Mediterranean. The one-time occurrence, signaling single occurrences, are the most widely dispersed across the map, indicating isolated incidents of oil spills. The geographical distribution shows clusters of high-frequency spill events in certain coastal zones, with a broader, more dispersed pattern of less frequent spills extending into the Eastern Mediterranean Sea.

### Potential sources of oil spill

**Figure 15:**
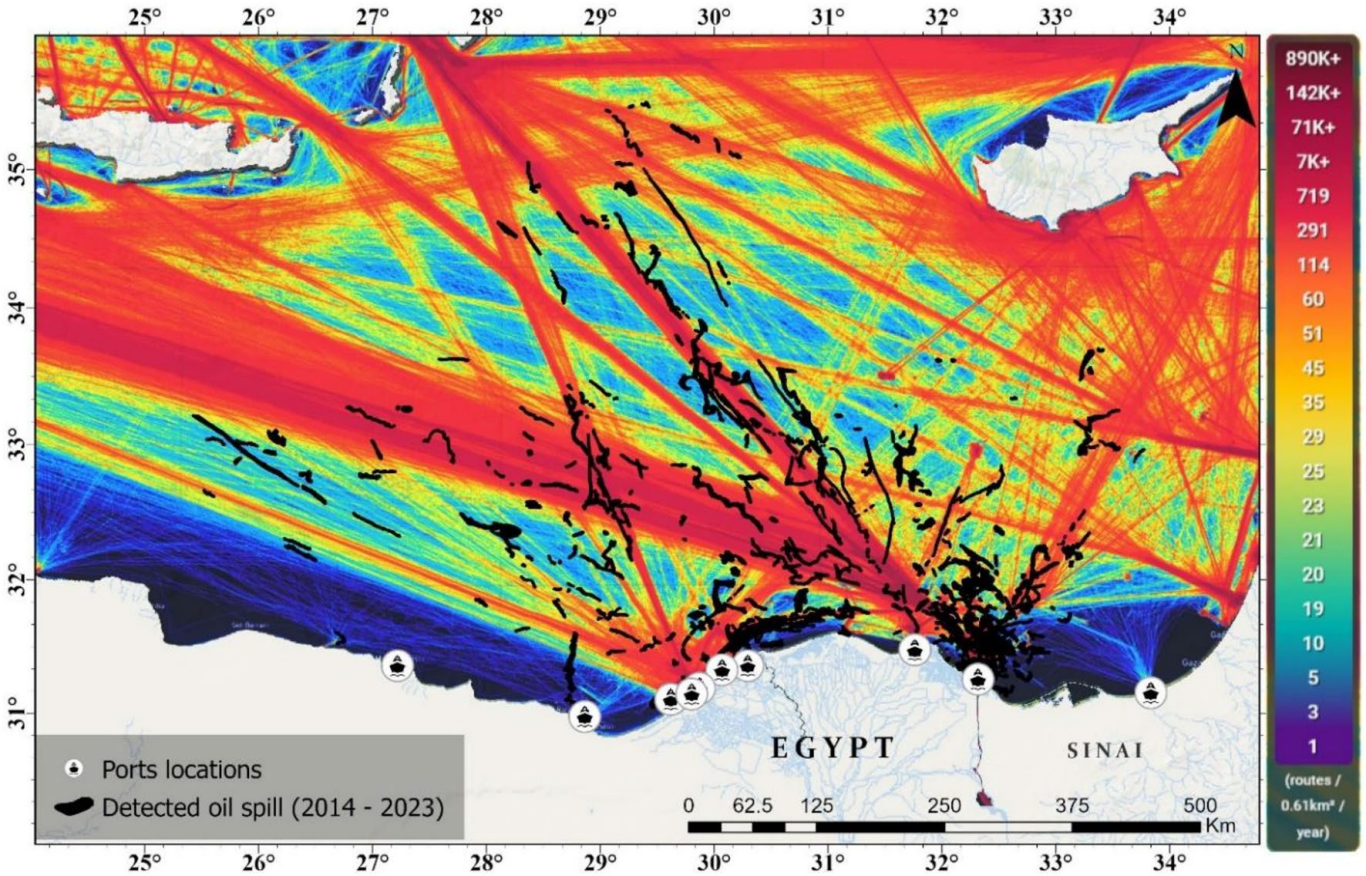
The relationship between the intensity of shipping ports and lanes with the locations where oil spills have been detected.

The geographical analysis of oil spill occurrences over a ten-year period, the northern entrance of the Suez Canal and the area north of the Nile Delta emerge as the most impacted regions. This heightened incidence of oil spills is closely associated with the shipping routes in and around the major ports like Port Said, Damietta, Alexandria, Idku, Abu Qir, Sidi Kerir, and El Dekheila (Fig. 15). On the other hand, ports such as Mersa el Hamra and Mersa Matroh have exhibited significantly lower oil spill frequencies, which can be attributed to their lesser shipping activities compared to the busier ports.

The geographical distribution of oil spills along the shipping routes, as depicted on the map, reveals a pattern where the majority of spills are located along the established marine traffic lanes that are indicated by the red and orange lines. These spills tend to form clusters around areas with the highest traffic density, particularly close to port entries and along the main shipping lanes that traverse the Eastern Mediterranean Sea. There is a notable spread of oil spills extending from the coastlines into the sea along these routes, indicating that spills are not confined to the immediate vicinity of the ports but are distributed along the length of the shipping paths.

**Figure 16:**
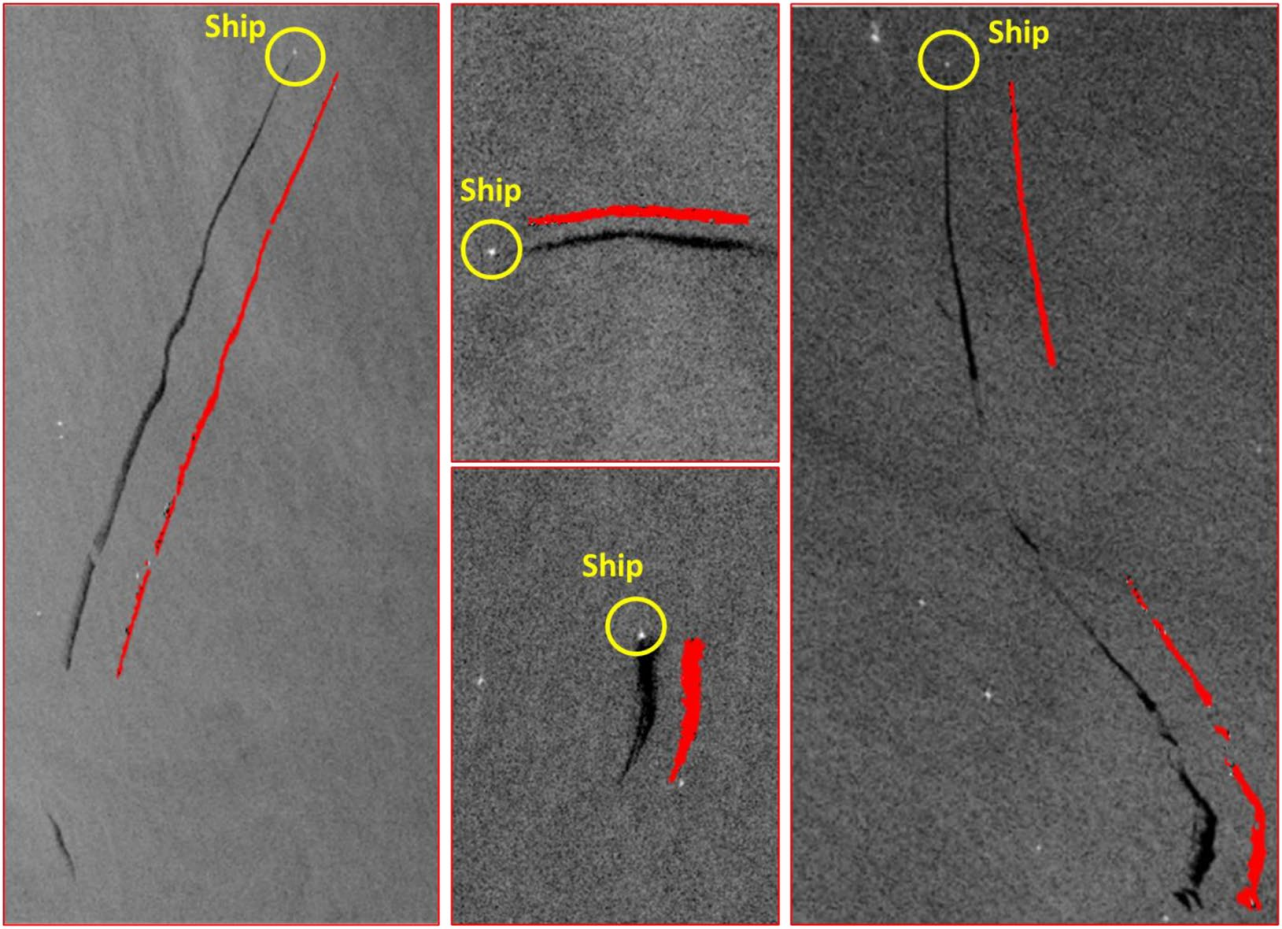
Detected ships illegally discharging oil and creating large oil spills, captured by SAR imagery.

The distribution is not uniform, with some segments of the routes, especially those closer to the coast, showing a denser accumulation of oil spill occurrences, while other parts, presumably those less trafficked, show a sparser presence of spills. A non-random distribution of oil spills appears, clearly associating with the ships (Fig. 16).

## Discussion

This decade-long study has methodically utilised Sentinel-1 SAR imagery to delineate a detailed pattern of oil spills, predominantly concentrated off the Egyptian coast, particularly near major ports and the Suez Canal. These findings are in alignment with those reported by El-Magd et al.^27^ and Kostianaia et al.^23^, underscoring a significant correlation between high maritime traffic volumes and increased pollution events. The marked concentration of oil spills around principal Egyptian ports such as Alexandria and Port Said, as well as along the vital conduit of the Suez Canal, underscores these areas as critical nodes where maritime commercial activities intersect prominently with environmental risk.

Our spatial–temporal analysis pinpoints the Egyptian (EEZ) and the vicinities surrounding key ports as critical hotspots for oil spill incidents, attributed largely to dense shipping activities. The fluctuations in oil spill frequency observed over the years, with significant spikes in 2017 and 2019, likely reflect variations in regional maritime traffic influenced by broader geopolitical and economic trends. These observations suggest an interplay between external maritime activities and the stringency of environmental regulation enforcement, as further evidenced by complementary findings from Zakaria et al.^29^ and Baghdady et al.^26^.

Seasonally, our study reveals a pronounced escalation in oil spill incidents during the summer and spring, coinciding with the peak maritime traffic associated with tourism and commercial shipping. This seasonal trend corroborates the observations made by Kostianoy and Lavrova^28^, highlighting that calmer sea conditions during these periods enhance the detection efficacy of SAR technology. In contrast, the more turbulent sea states in winter complicate the differentiation of anthropogenic oil spills from natural oceanographic features, a challenge also noted in the seminal works of Topouzelis et al.^16^ and Solberg et al.^30^.

Critically, the geographical proximity of detected oil spills—primarily within 10 to 92 km off the shoreline, especially near the northern entrance of the Suez Canal and the north Nile Delta—highlights areas where oil spills are likely to have immediate and deleterious effects on marine ecosystems. These findings align with Polinov et al.^31^, emphasizing the urgent need for effective monitoring and rapid response strategies in these proximal marine environments.

The deployment of SAR imagery in this research has been pivotal, providing high-resolution, real-time data that facilitates the swift identification and monitoring of oil spills across various environmental scenarios. However, the challenge remains to distinguish illegal discharges from natural seepage and to detect smaller spills that, cumulatively, can exert significant ecological impacts. Despite these challenges, the strategic utilization of SAR technology in our study underscores its invaluable role not only in immediate spill response but also in the development of predictive models and preventive strategies essential for the conservation of marine ecosystems and the maintenance of socioeconomic stability in regions dependent on maritime resources.

The findings of this study have substantial implications for environmental policy and maritime operations in the Mediterranean region. By identifying high-risk areas for oil spills, particularly around major ports and within the EEZ, our research provides critical insights that can inform the strategic deployment of monitoring resources and the development of targeted regulatory measures. Enhanced monitoring and stricter enforcement could not only help mitigate the risk of oil spills but also support the international maritime community in achieving compliance with environmental standards.

Despite the strengths of using SAR imagery for detecting oil spills, this approach has inherent limitations. The differentiation between oil spills and natural phenomena like algal blooms or look-alikes remains a challenge. Another limitation is the dependence on favorable weather conditions for optimal imaging, which can sometimes hinder the detection capabilities during adverse meteorological conditions. Future studies should focus on overcoming these limitations by integrating SAR imagery with other remote sensing technologies, such as multispectral and hyperspectral imaging, to improve the accuracy of oil spill detection and characterization. Research should also explore the potential of emerging technologies, such as machine learning algorithms, to distinguish between different types of spills and natural sea surface features more effectively. Moreover, longitudinal studies are needed to assess the long-term ecological impacts of oil spills, particularly in biologically sensitive regions.

## Conclusion

This study's analysis of oil spills in the Eastern Mediterranean over a decade, utilizing Sentinel-1 SAR imagery, has revealed a significant distribution of spills along the Egyptian EEZ, and concentration particularly near major ports and the Suez Canal, highlighting the link between heavy maritime traffic and environmental hazards. Temporal fluctuations, with notable spikes in certain years like 2017 and 2019 and seasonal increases in summer and spring, suggest a correlation between regional maritime traffic and the varying enforcement of environmental regulations. These spills are predominantly smaller in size, due to operational discharges from ships, emphasizing the cumulative impact of illegal oil discharges. Despite challenges in detection, SAR imagery has proven essential in monitoring and responding to these spills, underlining the need for enhanced monitoring, stricter enforcement, and international cooperation to mitigate oil pollution risks and protect marine ecosystems and dependent socio-economic activities in the Mediterranean.

## Recommendation

Given the study's findings, it is recommended that monitoring and surveillance be enhanced in the Eastern Mediterranean, particularly near major ports and along busy maritime routes, by employing advanced remote sensing technologies such as SAR imagery for the early detection of oil spills. The strict regulation and consistent enforcement of international maritime laws are crucial to prevent illegal discharges. Furthermore, collaborative efforts among regional governments, maritime authorities, and environmental organizations are essential for sharing best practices, resources, and information. Additionally, it is necessary to raise awareness and provide training for ship crews about the importance of environmental protection and the severe consequences of oil pollution to foster a culture of compliance and responsibility within the maritime industry. The need for vigilant monitoring is evident, emphasizing the requirement for aerial and satellite surveillance to detect and address oil spills promptly. These findings underscore the need to establish a robust system for identifying and prosecuting illegal discharges, integrating oil spill data with the ships' Automatic Identification System (AIS) data for efficient tracking.

## Data Availability

The necessary procedures to generate the data and the methodology have been outlined in the manuscript. The data that support the findings of this study are available from the corresponding author on reasonable request.
